# Central aortic hemodynamics following acute lower and upper-body exercise in a cold environment among patients with coronary artery disease

**DOI:** 10.1038/s41598-021-82155-x

**Published:** 2021-01-28

**Authors:** Heidi E. Hintsala, Rasmus I. P. Valtonen, Antti Kiviniemi, Craig Crandall, Juha Perkiömäki, Arto Hautala, Matti Mäntysaari, Markku Alén, Niilo Ryti, Jouni J. K. Jaakkola, Tiina M. Ikäheimo

**Affiliations:** 1 Center for Environmental and Respiratory Health Research (CERH), University of Oulu, P.O. Box 5000, 90014 Oulu, Finland; 2 Medical Research Center, University of Oulu and Oulu University Hospital, Oulu, Finland; 3 Centria University of Applied Sciences, Kokkola, Finland; 4 Research Unit of Internal Medicine, Medical Research Center Oulu, University of Oulu and Oulu University Hospital, Oulu, Finland; 5 Department of Internal Medicine, Texas Health Presbyterian Hospital, University of Texas Southwestern Medical Center and the Institute for Exercise and Environmental Medicine, Dallas, USA; 6 Cardiovascular Research Group, Division of Cardiology, Oulu University Hospital, University of Oulu, Oulu, Finland; 7 Finnish Defence Forces, Aeromedical Centre, Helsinki, Finland; 8 Department of Medical Rehabilitation, Oulu University Hospital and Center for Life Course Health Research, University of Oulu, Oulu, Finland

**Keywords:** Interventional cardiology, Coronary artery disease and stable angina, Randomized controlled trials, Lifestyle modification, Environmental impact

## Abstract

Exercise is beneficial to cardiovascular health, evidenced by reduced post-exercise central aortic blood pressure (BP) and wave reflection. We assessed if post-exercise central hemodynamics are modified due to an altered thermal state related to exercise in the cold in patients with coronary artery disease (CAD). CAD patients (n = 11) performed moderate-intensity lower-body exercise (walking at 65–70% of HR_max_) and rested in neutral (+ 22 °C) and cold (− 15 °C) conditions. In another protocol, CAD patients (n = 15) performed static (five 1.5 min work cycles, 10–30% of maximal voluntary contraction) and dynamic (three 5 min workloads, 56–80% of HR_max_) upper-body exercise at the same temperatures. Both datasets consisted of four 30-min exposures administered in random order. Central aortic BP and augmentation index (AI) were noninvasively assessed via pulse wave analyses prior to and 25 min after these interventions. Lower-body dynamic exercise decreased post-exercise central systolic BP (6–10 mmHg, p < 0.001) and AI (1–6%, p < 0.001) both after cold and neutral and conditions. Dynamic upper-body exercise lowered central systolic BP (2–4 mmHg, p < 0.001) after exposure to both temperatures. In contrast, static upper-body exercise increased central systolic BP after exposure to cold (7 ± 6 mmHg, p < 0.001). Acute dynamic lower and upper-body exercise mainly lowers post-exercise central BP in CAD patients irrespective of the environmental temperature. In contrast, central systolic BP was elevated after static exercise in cold. CAD patients likely benefit from year-round dynamic exercise, but hemodynamic responses following static exercise in a cold environment should be examined further.

Clinical trials.gov: NCT02855905 04/08/2016.

## Introduction

Regular exercise is a key component in the secondary prevention of coronary artery disease (CAD) patients, preventing the progress of the disease, as well as reducing the risk of myocardial infarctions or fatal cardiac events^1,2^.

As a health benefit, exercise administered acutely lowers blood pressure (BP) in healthy persons^3,4^. This effect has also been observed among persons with prehypertension and hypertension^5–7^ and among CAD patients^8^. Post-exercise hypotension (PEH) is known to occur after acute dynamic exercise^3,9,10^. In contrast, studies on static exercise have showed either reduced^5,11^ or increased PEH^12,13^. The timing of PEH varies depending on the type and intensity of the activity, but typically occurs within 120 min after exercise^14^. This BP lowering effect can persist for hours or a day^3,5^ and contributes to overall BP management^3,5^. Hence, with habitual physical activity a persistent lowering of BP is known to occur^15^.

Cold exposure increases cardiovascular strain due to vasoconstriction and related elevations in peripheral vascular resistance, blood pressure and cardiac workload^16–18^. Also, central aortic BP, reflective of the pressure load of the heart^19^, increases in a cold conditions^20–24^. Exercising in a cold environment increases cardiovascular strain further among CAD patients^17,25–27^. Also, recovery from exercise involving simultaneous cold exposure may differ from that performed in a thermoneutral environment. Specifically, the thermal state of subjects following exercise may affect PEH^28^, but the thermal responses after cold exposure or exercise are not well understood^29,30^. In addition, the exercise mode (i.e., dynamic or static) involving differing loads on the cardiovascular system (volume vs. pressure load)^31,32^ may be differentially influenced by cold exposure and affect post-exercise hemodynamics. For example, static exercise and cold exposure separately increase systemic vascular resistance, and a combination of both could result in absence of PEH. Overall, it is possible that repeated exposures to cold while exercising could dampen the long-term post-exercise lowering of BP.

The aim of the present study was to assess the separate and combined effects of lower or upper-body dynamic and static exercise in a cold environment on post-exercise central hemodynamics. We hypothesized that cold exposure, combined with dynamic lower and upper-body exercise, results in a lesser decrease in post-exercise central aortic BP and wave reflection compared with exercise in a neutral environment. Secondly, we hypothesized that static exercise would involve a higher central BP following exercise in a cold compared with a neutral environment.

## Results

### Lower-body aerobic exercise

**Figure 1:**
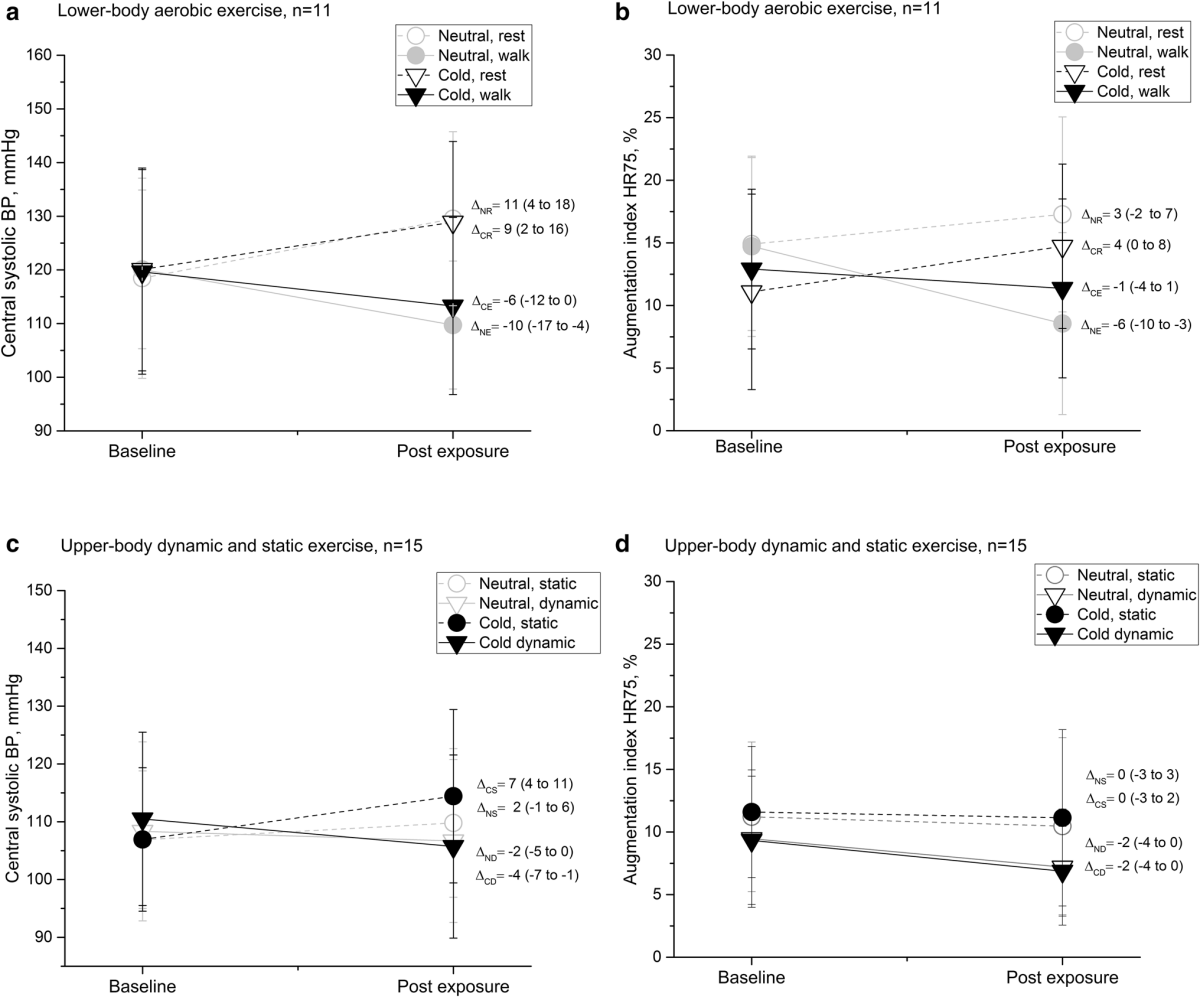
(**A**–**D**) Central aortic blood pressure (BP) and heart rate (HR) adjusted augmentation index (AI) before and 25 min after lower-body (a,b) and upper-body static and dynamic (c,d) exercise carried out at + 22 °C and − 15 °C. Graphs with group mean and standard deviations, delta values (∆) with group means and 95% coefficient of mean.

Mean T_sk_ decreased by 6.4 °C (p < 0.001) in the cold and 0.5 °C in the neutral conditions during the exercise intervention compared with pre-exposure baseline. Facial skin temperature decreased considerably from 30–31 to 11–14 °C (p < 0.001) both during rest and exercise in a cold environment. Post-intervention skin temperature recovered close to baseline after 25 min, with mean T_sk_ being 1 °C and facial skin temperature being 2–4 °C lower after exposure to cold conditions. At the end of the intervention the average whole-body thermal sensation of patients was: − 3/cold (cold rest), − 1/slightly cool (cold exercise), 0/neutral (neutral rest) and + 2/warm (neutral exercise). The achieved exercise intensity represented 69% and 66% of maximum HR at cold and neutral temperature, respectively. The rate of perceived exertion (RPE) varied from light to somewhat hard (11–14), both while exercising in a neutral and cold environment. Central hemodynamics after rest and lower-body aerobic exercise in cold and neutral temperatures are presented in Table 1 and Fig. 1. Exercise decreased central aortic systolic BP compared to rest, independent of the ambient temperature (decrease of − 6 to − 10 mmHg and increase of 9–10 mmHg after rest from baseline levels, p < 0.001). Of note, exercise decreased (p < 0.01) central aortic diastolic BP compared to rest, but only when performed in a neutral temperature (p = 0.047 for interaction between temperature, activity, and time). Brachial systolic and diastolic BP changes were comparable to the changes in central aortic BP. Post-exercise HR was elevated (p < 0.001), but with no effect of temperature. Concerning wave reflection, exercise decreased AI compared to rest, independent of temperature and with or without HR adjustments (ca. 8–40% decrease after exercise compared to baseline). Augmented pressure decreased and time to reflection increased following exercise compared to rest, independent of temperature. Concerning cardiac workload and perfusion, exercise decreased SEVR compared to rest, independent of temperature, however remaining > 100% for each measurement and patient.

**Table 1:** Central hemodynamics during rest and lower-body aerobic exercise in cold and neutral conditions (n = 11).

Variable	+ 22 °C				− 15 °C				p-values		
	Rest		Exercise		Rest		Exercise				
	Baseline	Post	Baseline	Post	Baseline	Post	Baseline	Post	T × t	a × t	T × a × t
**Blood pressure and heart rate**											
Central SBP, mmHg	118 (106, 131)	130 (119, 140)	120 (110, 130)	110 (102, 118)	120 (107, 133)	129 (119, 139)	119 (106, 132)	113 (102, 124)	0.74	< 0.001	0.38
Central DBP, mmHg	81 (73, 88)	88 (80, 96)	85 (76, 93)	80 (73, 87)	83 (76, 91)	87 (81, 93)	83 (75, 90)	84 (77, 91)	0.54	0.001	0.047
Brachial SBP, mmHg	129 (115, 142)	139 (127, 150)	131 (120, 141)	122 (114, 130)	133 (118, 148)	140 (128, 152)	131 (117, 145)	124 (112, 137)	0.89	< 0.001	0.60
Brachial DBP, mmHg	80 (73, 88)	87 (80, 95)	84 (75, 93)	79 (72, 86)	83 (76, 90)	86 (81, 92)	82 (75, 89)	83 (76, 90)	0.51	0.001	0.053
Heart rate, bpm	59 (55, 64)	55 (52, 59)	61 (56, 67)	67 (60, 73)	62 (57, 67)	56 (50, 62)	61 (56, 67)	67 (60, 73)	0.30	< 0.001	0.39
**Wave reflection**											
AI_HR75_, %	14 (9, 19)	18 (13, 24)	15 (10, 20)	9 (3, 14)	11 (4, 17)	16 (11, 21)	13 (9, 17)	12 (6, 16)	0.13	< 0.001	0.27
AI, %	22 (16, 29)	29 (22, 35)	22 (16, 27)	13 (5, 21)	17 (12, 22)	25 (20, 31)	19 (14, 24)	16 (11, 20)	0.09	< 0.001	0.55
AP, mmHg	9 (6, 13)	13 (8, 18)	8 (5, 11)	4 (2, 7)	7 (4, 10)	11 (8, 15)	8 (5, 11)	5 (3, 8)	0.51	< 0.001	0.92
P1, mmHg	112 (102, 121)	119 (110, 128)	113 (106, 121)	107 (102, 112)	115 (102, 127)	121 (111, 130)	112 (101, 122)	110 (100, 120)	0.42	< 0.001	0.42
P2, mmHg	121 (109, 133)	131 (121, 142)	121 (113, 129)	111 (106, 117)	122 (108, 136)	132 (121, 142)	120 (107, 132)	115 (104, 126)	0.38	< 0.001	0.52
Tr, ms	149 (140, 158)	146 (139, 154)	149 (140, 158)	154 (144, 164)	153 (144, 162)	146 (141, 152)	151 (145, 156)	151 (143, 158)	0.17	0.001	0.79
ED, ms	299 (283, 314)	318 (304, 332)	292 (276, 308)	283 (269, 298)	291 (282, 300)	309 (295, 323)	292 (279, 306)	288 (278, 298)	0.80	0.001	0.60
**Cardiac workload and myocardial oxygen supply/demand relation**											
cRPP, bpm × mmHg	7030 (6240, 7820)	7190 (6320, 8050)	7410 (6500, 8330)	7270 (6580, 7960)	7570 (6310, 8830)	7250 (6190, 8310)	7360 (6410, 8310)	7560 (6470, 8650)	0.76	0.44	0.16
bRPP, bpm × mmHg	7650 (6760, 8550)	7710 (6740, 8680)	8070 (7060, 9080)	8110 (7320, 8900)	8380 (6940, 9830)	7920 (6650, 9190)	8080 (6970, 9190)	8320 (7050, 9580)	0.53	0.20	0.27
SEVR, %	210 (193, 227)	214 (196, 233)	212 (193, 232)	197 (177, 216)	206 (182, 230)	219 (190, 248)	207 (186, 227)	193 (166, 220)	0.38	0.001	0.54

*T* temperature (cold vs. neutral), *t* time (baseline vs. post intervention), *a* activity (rest vs. exercise). The values represent means (95% CI).

*SBP and DBP* systolic and diastolic blood pressure, *AI*_*HR75*_ augmentation index adjusted to heart rate of 75 bpm, *AI* unadjusted augmentation index, *AP*_*HR75*_ augmented pressure adjusted to heart rate of 75 bpm, *AP* augmentation pressure, *P1 and P2* blood pressure at the first and second systolic pressure peak, *ED* ejection duration, *Tr* time to reflection, *cRPP and bRPP* central and brachial rate-pressure product, *SEVR* subendocardial viability ratio, *T* temperature (cold and neutral), *t* time (baseline and post intervention), *a* activity (rest and exercise).

P-values for interaction effects of two-way repeated measures ANOVA presented: *T* temperature (cold or neutral), *t* time (baseline or 25 min post exposure), *a* activity (rest or dynamic lower-body exercise).

### Upper-body static and dynamic exercise

Static exercise performed at cold temperature decreased mean T_sk_ by 3.8 °C (p < 0.001) compared with pre-exposure baseline. Facial skin temperature decreased considerably from 30–31 to 15–16 °C (p < 0.001). Skin temperature recovered close to the baseline values 25 min post cold exposure, with mean T_sk_ being 1 °C and facial T_sk_ being 2–3 °C lower after exposure to cold conditions. At the end of the intervention the average whole-body thermal sensation of patients was: − 2/cold (cold condition), and + 1/slightly warm (neutral condition). The achieved exercise intensity represented 46, 47, 48, 52, and 56% of HR_max_ in the neutral thermal conditions and 42, 43, 44, 47 and 50% in the cold intervention. RPE varied from fairly light to very hard (10 to 16) both while exercising in a neutral and cold environment. The post-exercise responses to static and dynamic exercise differed from each other for most parameters (e.g. central aortic BP main effect for exercise F = 22 and p < 0.001, interaction for exercise and temperature changes F = 8 ja p = 0.01), and therefore the results related to these are reported separately. Central aortic (p < 0.001) and brachial (p = 0.002) systolic BP increased following static exercise in cold conditions (Table 2, Fig. 1c,d). In neutral conditions, systolic BP remained unaltered. Post-exercise central and brachial DBP increased, but with no temperature effect. HR was lowered after static exercise regardless of thermal conditions. Concerning pulse wave reflection, both P1 (p = 0.001) and P2 (p < 0.001) were higher after exercising in the cold, and AI_HR75_ did not change. Concerning cardiac workload, SEVR increased after static upper-body exercise, independent of temperature.

**Table 2:** Central hemodynamics during graded upper-body static exercise in cold (− 15 °C) and neutral (+ 22 °C) conditions (n = 15).

	+ 22 °C		− 15 °C		p-value	
	Baseline	Post	Baseline	Post	T × t	t
**Blood pressure and heart rate**						
Central SBP, mmHg	107 (100, 114)	109 (102, 117)	107 (100, 114)	114 (106, 122)	0.02	neutral (ns), cold p < 0.001
Central DBP, mmHg	77 (72, 83)	80 (75, 85)	76 (71, 82)	81 (75, 86)	0.14	< 0.001
Brachial SBP, mmHg	118 (111, 126)	120 (111, 130)	119 (111, 126)	125 (117, 134)	0.04	neutral (ns), cold p = 0.002
Brachial DBP, mmHg	77 (71, 82)	79 (74, 85)	76 (70, 81)	80 (75, 86)	0.22	< 0.001
Heart rate, bpm	64 (57, 71)	61 (55, 67)	63 (59, 67)	58 (54, 61)	0.13	< 0.001
**Wave reflection**						
AI_HR75_, %	11 (8, 14)	11 (7, 15)	12 (9, 15)	12 (8, 16)	–	
AI, %	18 (13, 22)	19 (14, 23)	18 (14, 22)	20 (15, 25)	0.34	0.13
AP, mmHg	6 (4, 7)	6 (4, 8)	6 (4, 7)	7 (5, 10)	0.20	0.09
P1, mmHg	101 (95, 108)	104 (96, 111)	100 (94, 107)	106 (99, 114)	0.04	neutral (ns), cold p = 0.001
P2, mmHg	107 (100, 114)	110 (101, 118)	106 (99, 113)	114 (105, 122)	0.02	neutral (ns), cold p < 0.001
Tr, ms	146 (139, 153)	145 (141, 150)	146 (142, 149)	142 (137, 148)	-	
ED, ms	290 (281, 298)	292 (278, 305)	286 (277, 295)	293 (282, 304)	0.39	0.18
**Cardiac workload and myocardial oxygen supply/demand relation**						
cRPP, bpm × mmHg	6870 (6020, 7710)	6650 (5940, 7360)	6770 (6200, 7340)	6540 (6040, 7040)	0.95	0.12
bRPP, bpm × mmHg	7600 (6630, 8570)	7320 (6530, 8120)	7510 (6850, 8170)	7200 (6650, 7750)	0.88	0.10
SEVR, %	205 (191, 219)	218 (199, 236)	204 (195, 213)	224 (211, 237)	0.31	0.001

*T* temperature (cold vs. neutral), *t* time (baseline vs. post intervention). The values represent means (95% CI).

*SBP and DBP* systolic and diastolic blood pressure, *AI*_*HR75*_ augmentation index adjusted to heart rate of 75 bpm, *AI* unadjusted augmentation index, *AP*_*HR75*_ augmented pressure adjusted to heart rate of 75 bpm, *AP* augmentation pressure, *P1 and P2* blood pressure at the first and second systolic pressure peak, *ED* ejection duration, *Tr* time to reflection, *cRPP and bRPP* central and brachial rate-pressure product, *SEVR* subendocardial viability ratio, *T* temperature (cold and neutral), *t* time (baseline and post intervention), *a* activity (rest and exercise).

P-values for interaction effects of two-way repeated measures ANOVA presented: *T* temperature (cold or neutral), *t* time (baseline or 25 min post exposure).

Dynamic exercise performed at cold temperature decreased mean T_sk_ by 3.4 °C (p < 0.001) compared with pre-exposure baseline. Facial skin temperature decreased considerably from 30–31 to 15–16 °C (p < 0.001). Post-intervention skin temperature recovered close to the baseline after 25 min from cold exposure, with mean T_sk_ being 1 °C and facial T_sk_ being 2–3 °C lower after exposure to cold conditions. At the end of the intervention the average whole-body thermal sensation of patients was: − 1/slightly cool (cold dynamic), + 2/warm (neutral dynamic). The achieved exercise intensity represented 56, 62 and 73% of HR_max_ during dynamic exercise at the neutral temperature and 59, 66 and 80% of HR_max_ at the cold temperature. The RPE during dynamic exercise varied from fairly light to hard (11–15) at the neutral temperature and from somewhat hard to very hard (12–16) in the cold environment. Dynamic upper-body exercise decreased post-intervention central aortic systolic BP by approximately 2–4 mmHg (p = 0.01) from baseline (Table 3, Fig. 1c,d). Brachial BP was not altered. A higher post-exercise HR (7 bpm) was observed (p < 0.001), which was not temperature related. Concerning wave reflection, AI decreased after exercise, but the changes were not significant after adjusting for HR. Also, P2 decreased (p < 0.01) following exercise, but with no temperature effect. Parameters reflecting cardiac workload demonstrated increased central and brachial RPP and decreased SEVR following exercise, at both temperatures. SEVR remained at > 100% for each measurement and patient.

**Table 3:** Central hemodynamics during upper-body dynamic exercise in cold (− 15 °C) and neutral (+ 22 °C) conditions (n = 15). The values represent means (95% CI).

Variable	+ 22 °C		− 15 °C		p-value	
	Baseline	Post	Baseline	Post	T × t	t
**Blood pressure and heart rate**						
Central SBP, mmHg	107 (99, 115)	105 (98, 112)	107 (99, 114)	103 (96, 110)	0.34	0.01
Central DBP, mmHg	78 (72, 83)	79 (74, 84)	76 (71, 81)	76 (70, 82)	0.45	0.75
Brachial SBP, mmHg	119 (110, 128)	118 (109, 127)	119 (110, 127)	116 (108, 124)	0.19	0.33
Brachial DBP, mmHg	77 (72, 82)	78 (73, 83)	76 (71, 80)	75 (69, 81)	0.44	0.81
Heart rate, bpm	61 (58, 65)	68 (64, 73)	61 (58, 64)	68 (64, 71)	0.79	< 0.001
**Wave reflection**						
AI_HR75_, %	9 (6, 13)	8 (5, 10)	10 (7, 13)	7 (5, 9)	–	
AI, %	16 (12, 20)	11 (7, 15)	16 (14, 19)	11 (8, 13)	0.93	< 0.001
AP, mmHg	5 (3, 6)	3 (2, 4)	5 (4, 6)	3 (2, 4)	0.82	< 0.001
P1, mmHg	101 (94, 107)	101 (94, 107)	101 (94, 108)	99 (92, 106)	0.33	0.27
P2, mmHg	105 (98, 113)	104 (97, 110)	106 (98, 113)	102 (94, 109)	0.36	< 0.01
Tr, ms	151 (144, 157)	149 (143, 155)	150 (146, 154)	153 (148, 157)	–	
ED, ms	292 (284, 300)	274 (262, 287)	293 (284, 302)	279 (269, 288)	0.63	< 0.001
**Cardiac workload and myocardial oxygen supply/demand relation**						
cRPP, bpm × mmHg	6560 (6010, 7100)	7170 (6500, 7830)	6510 (6020, 7000)	6930 (6390, 7480)	0.39	0.001
bRPP, bpm × mmHg	7270 (6610, 7930)	8070 (7210, 8930)	7230 (6660, 7800)	7810 (7170, 8460)	0.45	0.001
SEVR, %	207 (194, 220)	196 (181, 211)	205 (193, 218)	192 (182, 203)	0.70	< 0.001

*SBP and DBP* systolic and diastolic blood pressure, *AI*_*HR75*_ augmentation index adjusted to heart rate of 75 bpm, *AI* unadjusted augmentation index, *AP*_*HR75*_ augmented pressure adjusted to heart rate of 75 bpm, *AP* augmentation pressure, *P1 and P2* blood pressure at the first and second systolic pressure peak, *ED* ejection duration, *Tr* time to reflection, *cRPP and bRPP* central and brachial rate-pressure product, *SEVR* subendocardial viability ratio, *T* temperature (cold and neutral), *t* time (baseline and post intervention), *a* activity (rest and exercise).

P-values for interaction effects of two-way repeated measures ANOVA presented: *T* temperature (cold or neutral), *t* time (baseline or 25 min post exposure).

## Discussion

This study generally found favorable effects of acute moderate-intensity dynamic exercise on post-exercise central hemodynamics in patients with stable CAD, independently of the ambient temperature in which the exercise was performed. Lower-body dynamic exercise elicited the most significant central BP lowering effect. On the other hand, upper-body static exercise in the cold increased post-exercise central systolic BP.

We hypothesized that exercise in a cold environment would involve higher vascular resistance, which effects would continue following the cessation of exercise and abolish the post-exercise reduction in arterial blood pressure^28^. In contrast to our hypothesis, we observed similar post-exercise lowering of central systolic BP and wave reflection after acute lower (brisk walking) or upper-body (arm-ergometer) dynamic exercise in both cold and neutral temperatures. Central systolic BP lowered by 6–10 mmHg after acute lower-body dynamic and by 2–4 mmHg after upper-body dynamic exercise. To our knowledge, there are no previous studies examining the combined effects of cold and exercise on post-exercise central BP in CAD patients. To date, previous research has assessed central aortic BP in cold only under resting conditions^20–23,33,34^. Our finding of a reduced post-exercise central systolic BP among CAD patients after exercise is in accordance with studies concerning healthy, as well as prehypertensive and hypertensive persons^3,7,35^. We also detected comparable post-exercise BP responses for central aortic and brachial BP, which is consistent with our findings for exposure to cold at rest^22^.

A similar post-exercise reduction in central aortic systolic BP after exercise, regardless of environmental temperature, could be due to a few reasons. Dynamic exercise involves increased blood flow to the working muscles and accompanying vasodilation^9^. This response could offset peripheral cold-induced vasoconstriction during exercise in the cold and enable quick post-exercise recovery. However, our previous study from the same data showed that brachial systolic BP remained higher throughout the exercise intervention in cold compared with a neutral environment^26^. Instead, we suggest that the similar post-exercise response could be due to a rapid withdrawal of sympathetic and increase in cardiac vagal activity immediately after exercise at both temperatures^8^, quickly reducing BP to comparable post-exercise levels. In addition, the lowered T_sk_ during exercise in the cold had almost returned to baseline 25 min after exercise. Though, the elevated post-exercise HR implies that some sympathetic influence prevailed.

We hypothesized that static exercise would involve a higher central BP following exercise in a cold compared with a neutral environment. Supporting our hypothesis, we observed a higher post-exercise central systolic and diastolic BP after static upper-body exercise in cold (arm-crank) compared with neutral conditions. The observed post-exercise increase in central BP and arterial stiffness are consistent with other studies involving static exercise conducted at neutral environmental temperatures^13^. However, some studies have also demonstrated PEH^5,11^ and the different findings could relate to the employed exercise intensity and involved muscle groups. To our knowledge, there is only one previous study that assessed the effects of whole-body cold exposure and static exercise on central aortic BP among healthy men^24^. They observed elevated central aortic BP and wave reflection when static handgrip was applied in cold compared with neutral conditions. However, their follow-up after the intervention was 3 min, which probably is not long enough for detecting recovery responses^24^.

The reason for the higher central BP after static exercise in a cold environment could be due to a few factors. Static exercise itself is related with increased sympathetic activity and pressor response due to mechanical compression, reduced perfusion, accumulation of metabolites and muscle chemoreflex activation^31,32^. Also, whole-body cold exposure increases sympathetic activation and vascular resistance^16,17^. Their combination can further increase cardiac workload among CAD patients^25^ and affect post-exercise recovery of BP. Together with a higher post-exercise central BP, we also observed a lowered HR. This response is consistent with previous reports and is related to the coactivation of both sympathetic and vagal activity during static exercise^36^. Facial exposure to cold also increases vagal activity^17,37^ and could further reduce post-exercise HR.

The beneficial blood pressure lowering effects of acute exercise may persist even for a day and play an important role in overall BP management^3,5^. This cardioprotective effect becomes even more significant with habitual exercise^15^. Regular physical activity is essential in the treatment and secondary prevention of CAD patients^1,2^. On the other hand, exercise at low environmental temperatures involves augmented cardiovascular strain and emphasized in CAD involving a myocardial blood flow limiting disease^17,25,26^. Especially patients with obstructive coronary stenoses, and who are inhaling cold air while exercising, show responses favoring myocardial ischemia^27^. The blunting of PEH could become more pronounced among CAD patients exercising repeatedly in a cold environment. However, our study suggests that acute dynamic lower- and upper-body exercise reduces systolic BP and wave reflection to a similar extent for exercise at cold and neutral ambient temperatures. Secondly, systolic BP recovers normally after moderate-intensity lower-body aerobic exercise in cold. Thirdly, we observed an increased systolic BP after acute static upper-body exercise in cold, indicating a slower recovery of post-exercise BP. Therefore, it remains to be established whether static exercise in the cold is beneficial in long-term BP management. Examining the joint effects of exercise and cold exposure on cardiovascular responses among persons with an ischemic heart disease help understanding their benefits and potential health risks^38,39^.

The strengths of the study include strictly controlled level of thermal exposure and exercise. Furthermore, each subject served as his own control, therefore eliminating confounding due to intra-individual factors. In addition, randomization of the trials limits an order effect. Finally, strict selection of participants helps reducing confounding from other causes than those related to cardiovascular diseases. As a limitation, including a longer follow-up of repeated central BP measurements could have further confirmed our findings. Restricting the participants only to those with stable CAD, does not enable distinguishing the observed responses from other disease states or healthy persons. For safety reasons, we did not cease medication of the patients during the experiments. Hence, we evaluated cardiovascular responses of individuals who are being treated for CAD, rather than examining the disease in the absence of medical treatment.

### Conclusions

These results show that post-exercise lowering of central aortic BP among stable CAD patients is largely unaffected by the environmental temperature where the dynamic exercise is performed. In contrast, systolic BP was elevated after static upper-body exercise in cold conditions, which provides a foundation for further investigations into the mechanisms of this response. Overall, this information encourages stable CAD patients to perform year-round exercise, such as walking. Future studies should explore the use of exercise as a potential adjunct treatment in CAD, and where also environmental temperature must be taken under consideration.

## Methods

The study design, recruitment and intervention protocols, and thermal measurements have also been described in our previous publications^26,40^. The study consisted of patients with stable CAD that had been treated at the Oulu University Hospital because of non-ST-elevation myocardial infarction (Table 4). The patient recruitment took place 10-12/2015 and 10-12/2016 and the controlled measurements were performed at Kastelli Research Center Oulu, Finland. The inclusion criteria consisted of a diagnosed of CAD (Canadian Cardiac Society [CCS] class I–II, a non-ST-elevation myocardial infarction occurring at least 3 months ago, male and age from 40 to 70 years. The exclusion criteria were: CCS class III–IV, chronic atrial fibrillation, claudication, unstable angina pectoris, left ventricular ejection fraction less than 40%, a history of coronary artery bypass grafting, pacemaker, significant ECG abnormalities during rest, presence of doctor-diagnosed asthma or diabetes and current smoking. Clinical exercise tests were performed to assess maximal exercise capacity and to detect possible ECG abnormalities, with these findings described elsewhere^26,41^. Prior to the experiments, body composition was assessed by bioimpedance measurements (InBody720 Biospace, Seoul, Korea). The subjects also completed a questionnaire inquiring about their perceived health, physical activity at work and during leisure time, physical fitness and use of alcohol. The study subjects were given both oral and written information of the study and an informed consent was required for participation. The study was conducted in accordance with the declaration of Helsinki and was approved by the Ethics Committee of the Northern Ostrobothnia Hospital District, Oulu, Finland. All patients provided informed consent to participate to the study. The study was registered at ClinicalTrials.gov (NCT02855905, 04/08/2016).

**Table 4:** Characteristic of the study population.

Variables	Lower-body aerobic exercise n = 11	Upper-body dynamic and static exercise n = 15
Age, years	57.6 (7.0)	59.4 (8.1)
Body mass index, kg/m^2^	29.6 (6.3)	27.9 (4.0)
Body fat, %	25.3 (9.3)	23.0 (6.2)
Peak oxygen consumption, mL/kg/min	29.9 (6.3)	31.5 (5.8)
Systolic blood pressure, mmHg	128 (19)	120 (14.5)
Diastolic blood pressure, mmHg	82 (9)	76 (11.2)
Hypertension, n	7 (64%)	14 (93%)
Time elapsed from MI, months	15 (5)	27 (10)
Single vessel disease	9 (82%)	10 (67%)
Double vessel disease	1 (9%)	4 (27%)
Triple vessel disease	1 (9%)	1 (7%)
Number of stents	2 (varied 1 to 5)	2 (varied 0 to 5)
Ejection fraction	60 (9)%	64 (7)%
**Medication**		
Acetylsalicylic acid, n	10 (91%)	15 (100%)
Beta-blockers, n	6 (55%)	11 (73%)
Statins, n	9 (82%)	13 (87%)
Angiotensin converting enzyme inhibitors, n	6 (55%)	6 (40%)
Angiotensin receptor blockers, n	3 (27%)	4 (27%)
Calcium channel blockers, n	2 (18%)	3 (20%)
**Health status, n**		
Excellent	1 (9%)	4 (27%)
Quite good	4 (36%)	6 (40%)
Average	6 (55%)	5 (33%)
Quite poor/very poor	0 (0%)	0 (0%)
Use any alcoholic drinks (even occasionally), n	9 (82%)	15 (100%)
**Physical demands of work, n**		
Mainly sitting	5 (45%)	11 (73%)
Much walking	2 (18%)	1 (7%)
Much waking and lifting	3 (27%)	3 (25%)
Heavy manual labor	1 (9%)	0 (0%)
**Leisure-time physical activity, n**		
Never	1 (9%)	0 (0%)
Rarely	7 (64%)	10 (67%)
Often	2 (18%)	4 (27%)
Very often	1 (9%)	1 (7%)
**Physical fitness status, n**		
Excellent	1 (9%)	2 (13%)
Quite good	7 (64%)	8 (53%)
Average	3 (27%)	5 (33%)
Quite poor/very poor	0 (0%)	0 (0%)

Values are the number of the patients or means ± standard deviation.

*BMI* body mass index, *BF* Body fat percentage, *Peak VO2* estimated (3.5 × MET) symptom-limited maximal oxygen uptake, *SBP* resting systolic blood pressure, *DBP* resting diastolic blood pressure.

### Study design

#### Lower-body aerobic exercise

**Figure 2:**
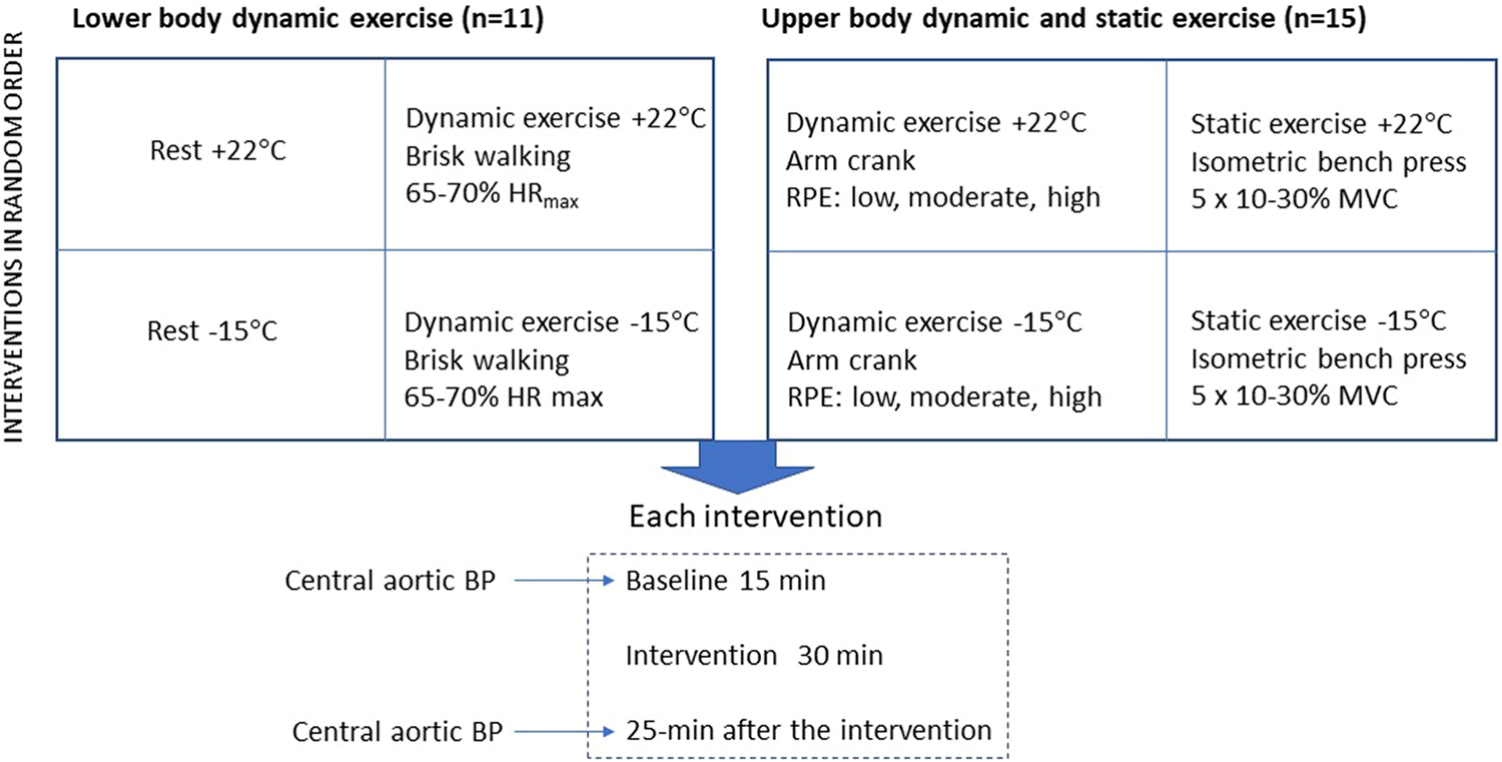
Study design describing the lower and upper-body exercise tests conducted in a warm (+ 22 °C) and cold (− 15 °C) environment.

Each patient (n = 11) (Table 4) participated to four different interventions in a climatic chamber, each administered in random order (Fig. 1). These conditions were rest at cold (− 15 °C) and neutral (+ 22 °C) conditions, and 30 min of lower-body exercise in both thermal conditions (Fig. 2). The exercise consisted of moderate-intensity walking on a treadmill, which intensity corresponded to the recommended intensity and duration of health-enhancing aerobic exercise^42^. The walking speed was adjusted based on target heart rate (HR) and calculated as: resting HR + 0.45 × [peak HR − resting HR] corresponding to moderate exercise intensity, the heart rate being approximately 65–70% of peak HR. Walking speed remained constant for each subject while exercising in cold and neutral conditions.

#### Upper-body static and dynamic exercise

Each patient (n = 15) (Table 4) participated to four different interventions in a climatic chamber, with each performed in random order. These were static upper-body exercise at cold (− 15 °C) and neutral (+ 22 °C) conditions, and dynamic upper-body exercise in the same conditions, each for 30 min (Fig. 2). The static upper-body exercise consisted of 5-min pre-exposure followed by five 1.5-min static contraction against a bench press with different intensities (Newtest Leg Force [bench press mode], Newtest, Oulu, Finland), performed at 10%, 15%, 20%, 25% and 30% of maximal voluntary contraction (MVC)^40^. MVC was determined as the largest of three bench press contractions aiming to maximal force and measured approximately one hour before the first trial. Participants had a 4-min break following each session. The dynamic upper-body exercise consisted of 5-min pre-exposure followed by three 5-min exercise periods of arm cranking exercise (Monark 881E, Vansbro, Sweden) at different intensities. The intensity of exercise was adjusted to mild-, moderate and high and based on subjective ratings of Perceived Exertion (11–12 fairly light as a mild-, 13–14 somewhat hard as a moderate- and 15–16 hard as a high-intensity), with the identical workloads performed between thermal conditions. Subjects had 4 min rest between each dynamic exercise bout.

#### Employed temperature exposure and exercise

The employed cold temperature (− 15 °C, 1.0 m/s) was selected to simulate conditions encountered often in the northern hemisphere during the winter. Similarly, the administered clothing represented that typically worn during the winter where cold exposure is largely targeted to the face. For the upper-body exercise, the insulation value of the clothing ensemble was 2.13 clo and consisted of underwear, insulated trousers and jacket, overtrousers and jacket, socks and shoes. Clothing insulation was reduced for lower-body exercise (1.88 clo) in the cold and was further reduced for this bout in neutral conditions (0.75 clo) to avoid heat strain. Each experiment trial was separated by at least one week. The patients participated in the experiments at the same time of the day. They were instructed to avoid heavy exercise 24 h, use of alcohol 48 h and coffee/caffeine related beverages 2 h prior to the experiments. They were also asked to continue their normal use of medication^26,40^.

### Measured parameters

#### Central aortic BP

We have previously described the protocol for assessing central aortic BP in our publication reporting cold-related responses in hypertensive subjects^22,29^. Central aortic BP was assessed non-invasively via radial artery applanation tonometry (SPC-301, Millari Instruments, Houston TX, USA) by the same operator during baseline before and 25 min after each intervention. The measurements were performed in seated position in neutral ambient temperature (+ 22 °C). Measured radial artery pressure waves were calibrated with brachial BP values (BP 200+, Schiller, Baar, Switzerland). Thereafter, central aortic BP was computed by using a build-in mathematical algorithm^43^ (SphygmoCor Px,AtCor Medical, Sydney, Australia). Central systolic, diastolic, and pulse pressure were defined from the pressure curve. Cardiac workload was estimated with central and brachial rate-pressure product (RPP) computed as product of systolic BP (mm Hg) and HR (bpm). Augmentation index (AI), an index of wave reflection^44^, was computed as the difference between second and first systolic pressure peaks, i.e., the augmented pressure, divided by the pulse pressure: (P2 − P1)/PP. Wave reflection describes the pressure wave reflected backwards toward aorta from more distal parts of the arterial tree, increasing left ventricular load when occurring during systole^44^. AI is inversely dependent on HR, and therefore was adjusted to a HR of 75 bpm^45^. Both adjusted and non-adjusted indexes are presented. Reflection time (Tr), the time that the pressure wave needs to reach the main reflection site and return^44^, was defined as the time between the onset of the pulse waveform and the onset of the reflected systolic central waveform. Subendocardial viability ratio (SEVR) was computed as a central aortic BP/time integral ratio of systolic and diastolic phases and provides an estimate for myocardial oxygen supply/demand relation^46^. Data quality was ensured by rejecting measurements via a built-in quality control operator index (Sphygmocor Px) below 75% (i.e., ensuring low variation for pulse height, diastolic pressure, and shape of the pressure wave during systole). The measured average operator indexes were 94 ± 5% (mean ± SD) for the first (lower-body aerobic exercise) and 92 ± 6% for the second (upper-body dynamic and static exercise) study protocol.Brachial BP was measured at 5-min intervals (Schiller BP 200+, Baar, Switzerland) during baseline, intervention and follow-up for comparison and to calibrate simultaneously measured central aortic BP. Ratings of perceived of exertion was obtained throughout exercise^47^. HR was measured continuously by a 12-lead ECG (Cardiosoft V6.71, GE Healthcare, Freiburg, Germany).

### Thermal responses

Skin temperature was measured continuously using thermistors (NTC DC95, Digi-Key, Thief River Falls, MN, USA) attached to the right scapula, left cheek, forehead, left calf, right anterior thigh, dorsal side of left index finger (middle phalanx), left hand, left forearm, right shoulder, left upper chest^26,40^. Data were recorded at 20 s intervals with two temperature data loggers (SmartReaderPlus,Acr Systems Inc., BC, Canada). Mean skin temperature (T_sk_) was calculated as follows: T_sk_ = ∑ki × tski = [0.07 × forehead + 0.175 × right scapula + 0.175 × left upper chest + 0.07 × right arm + 0.07 × left arm + 0.05 × left hand + 0.19 × right anterior thigh + 0.2 × left calf]^48^. Thermal sensations were inquired using scales of perceptual judgements on personal thermal state^49^.

### Statistical analyses

We conducted a sample size estimation and power analysis (G-Power 3.1.0) prior to the study which estimated to detect statistically significant differences in BP between a warm and cold environment [Power (1-ß err prob), 0.9, Cohen’s effect size 0.8, α err prob 0.05] with 15 participants. Normal distribution of the analyzed parameters was confirmed with Shapiro–Wilks-tests. First, we used a 3-way ANOVA to assess global effects of exercise, temperature and time using within-subjects factors time (baseline vs. post intervention), temperature (cold vs. neutral) and activity (exercise vs. rest). Based on the observed interaction for exercise, we conducted separate 2-way ANOVAs for static and dynamic upper-body exercise interventions where the main effects of temperature (cold vs. neutral) and time (baseline vs. post-intervention) were compared. For any observed interaction, separate post hoc-analyses were carried out to compare means between the temperature conditions. The results are expressed as means and their standard deviations (SD) or as confidence intervals (CI). Statistical significance was set at p < 0.05. Statistical analyses were performed with IBM SPSS for Windows version 23 (IBM Corp, Armonk, NY, USA).

## Abbreviations

AI: Augmentation index
BP: Blood pressure
CAD: Coronary artery disease
HR: Heart rate
PEH: Post-exercise hypotension
RPE: Rate of perceived exertion
RPP: Rate pressure product
SEVR: Subendocardial viability ratio
T_sk_: Mean skin temperature
